# Simultaneously Enhancing Spectral Resolution and Sensitivity in Heteronuclear Correlation NMR Spectroscopy**

**DOI:** 10.1002/anie.201305709

**Published:** 2013-09-06

**Authors:** Liladhar Paudel, Ralph W Adams, Péter Király, Juan A Aguilar, Mohammadali Foroozandeh, Matthew J Cliff, Mathias Nilsson, Péter Sándor, Jonathan P Waltho, Gareth A Morris

**Affiliations:** School of Chemistry, University of ManchesterOxford Road, Manchester, M13 9PL (UK); Institute of Organic Chemistry, Hungarian Academy of SciencesPusztaszeri út 59–67, 1025 Budapest (Hungary); Department of Chemistry, Durham UniversitySouth Road, Durham, DH1 3LE (UK); Manchester Institute of Biotechnology131 Princess Street, Manchester, M1 7DN (UK); Department of Food Science, University of CopenhagenRolighedsvej 30, 1958 Frederiksberg C (Denmark); Agilent Technologies R & D a. Marketing GmbH & Co. KGHewlett–Packard Strasse 8, 76337 Waldbronn (Germany)

**Keywords:** bilinear rotation decoupling, gHSQC, homonuclear decoupling, NMR spectroscopy, structure elucidation

A method for acquiring pure shift heteronuclear single quantum correlation (HSQC) NMR spectra in real time is described. A windowed acquisition scheme consisting of trains of bilinear rotation decoupling (BIRD)^1^, ^2^ refocusing elements is used to acquire chunks of data with refocused *J*_HH_ modulation while suppressing *J*_XH_ with broadband heteronuclear decoupling. The resultant spectra show both enhanced resolution in *F*_2_ and enhanced signal-to-noise ratio.

Scalar spin–spin (*J*) coupling provides valuable information for molecular structure elucidation, but the multiplet structure it causes is very expensive in terms of spectral resolution. In ^1^H NMR spectroscopy, multiplets are often many times the width of a single line. It is routine to suppress heteronuclear couplings (*J*_XH_) by broadband decoupling,^3^–^7^ but only recently have experimental methods for homonuclear broadband decoupling become practical. These “pure shift” or “chemical-shift resolved” or “δ-resolved” methods^8^–^19^ can give resolution improvements approaching an order of magnitude, far in excess of any gains to be realistically expected from increases in the static magnetic field. However, all of these methods suffer to a greater or lesser extent from reduced sensitivity compared to conventional measurements. Here we describe an experimental method for obtaining pure shift heteronuclear single quantum correlation (HSQC) spectra, in which real-time homodecoupling using the BIRD pulse sequence element^1^ leads to the first simultaneous resolution and signal enhancement in the directly detected (^1^H) dimension. (Homodecoupling has previously been described for the HSQC experiment, but only in the indirect (^13^C) dimension.^20^)

The HSQC experiment is the most widely used NMR method for correlating the chemical shifts of directly-bonded ^13^C–^1^H pairs. In its conventional^21^ form, it shows proton multiplet structure in *F*_2_, which limits resolution in the spectra of complex species. It has recently been shown^17^, ^22^, ^23^ that it is possible to extend the pure shift methods currently used, which rely on stitching together separate measurements of short periods of decoupled signal, to real-time acquisition, in which homonuclear couplings are periodically refocused, by applying appropriate spin manipulations during the acquisition of a single free-induction decay. Such J-refocusing sequence elements are generally designed to be broadband, as distinct from classical selective^24^, ^25^ or band-selective^26^ homodecoupling; in the case of HSQC, J-refocusing uses a BIRD pulse sequence element and a hard (nonselective) 180° pulse. The BIRD sequence element,^1^ which, as its name suggests, was originally intended for broadband homonuclear decoupling, has, until recently,^12^ been used almost exclusively for decoupling in the indirect dimension of heteronuclear 2D experiments.^27^ Here, the combined effect of the BIRD sequence and the hard 180° pulse is to invert only those protons *not* directly coupled to ^13^C, thus refocusing the effects of couplings between the latter protons and protons that *are* directly coupled (bonded) to ^13^C and whose signals are recorded in HSQC. The great advantage of the BIRD method here is that, in contrast to Zangger–Sterk type methods,^8^, ^9^, ^22^, ^23^ it incurs no extra sensitivity penalty; indeed, the sensitivity is generally increased.

The BIRD sequence element has already been very effectively used to obtain pure shift ^1^H-^13^C HSQC spectra,^16^ and pure shift 1D proton spectra of strongly coupled species.^12^ In both cases, the pure shift dimension was constructed from multiple separate acquisitions of short chunks of data, requiring ancillary software for the generation of decoupled spectra. Here we demonstrate how pure shift HSQC data with comparable resolution may be obtained much more quickly (to the point where a pure shift spectrum can require less time to acquire than a conventional spectrum) and without the need for any extra data processing. The one restriction is that the nucleus observed indirectly, generally ^13^C, should not itself show homonuclear coupling; thus, for example, the proposed sequence is not suitable for fully ^13^C-labeled compounds.

The pulse sequence used is shown in Figure 1. The initial part of the sequence is a conventional gHSQC,^21^ with the double insensitive nuclei enhanced by polarization transfer (INEPT) followed by a windowed data acquisition, in which the effects of homonuclear coupling are periodically refocused. Applying *n* BIRD/180° J-refocusing elements during the acquisition time (*at*) results in a free induction decay built up of an initial chunk of data of duration *at*/2*n*, (*n*−1) chunks of duration *at*/*n*, and a final chunk of *at*/2*n*. Provided that *n*≫(*at*×*J*_HH_), evolution under the homonuclear scalar coupling can be neglected, although care is needed to ensure that chemical shift evolution is accurately refocused during the J-refocusing element. More frequent J-refocusing gives cleaner spectra, but at the expense of some extra line broadening owing to imperfect refocusing and T_2_ relaxation. The BIRD real-time acquisition scheme differs slightly in timing from that previously proposed,^17^ requiring fewer J-refocusing elements for a given spectral quality. Heteronuclear couplings are suppressed as usual by broadband irradiation (denoted CPD in Figure 1); the intermittent nature of the decoupling limits the types of modulation favored. Because BIRD selects protons directly bonded to ^13^C, one class of coupling is not refocused, that between geminal protons. Spectra thus show singlet signals for all ^1^H sites except for nonequivalent methylene protons, for which doublet signals are seen (full details of the sequence are given in the Supporting Information).

**Figure 1:**
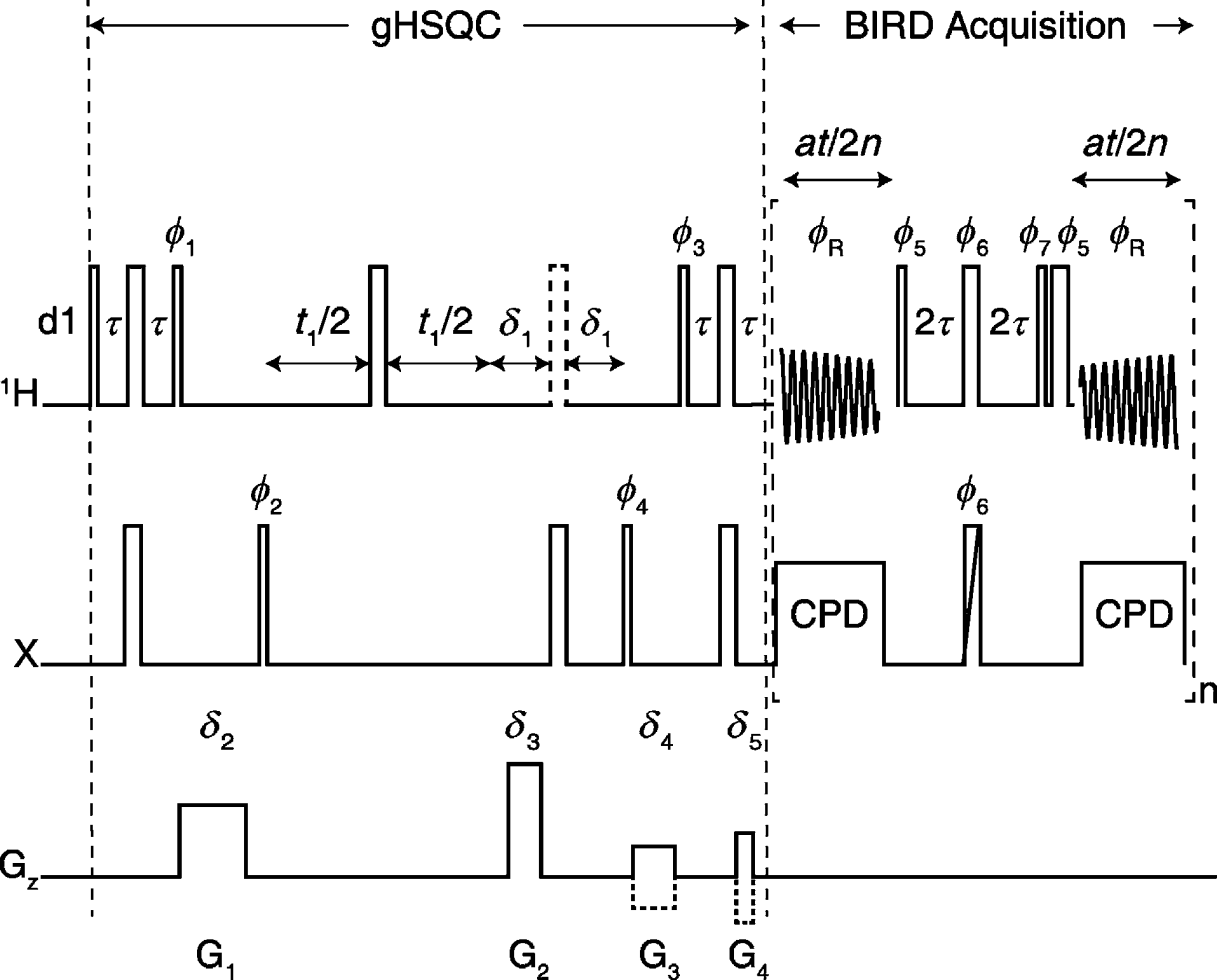
Pulse sequence for real-time pure shift gHSQC using BIRD. Narrow rectangles are 90° RF pulses, wide are 180° pulses, and wide with a diagonal line are either hard 180° pulses or composite 180° pulses. Gradient pulses G_1_−G_4_ follow the normal pattern for gHSQC, and *τ*=1/(4^1^*J*_XH_). The dotted proton RF pulse (0–2 times the duration of 90° pulse) centered between *δ*_1_ delays is for multiplicity editing; for edited spectra this pulse is 180° and *δ*_1_=2*τ*, which causes methylene protons to appear with opposite phase to methine and methyl; for unedited spectra this pulse is removed and *δ*_1_ is set to *δ*_3_ plus associated stabilization delay. The second *δ*_1_ delay precedes a delay equivalent to a hard proton 180° pulse, which compensates for the evolution during the 180° pulse in middle of the t_1_ evolution. Each BIRD/180° J-refocusing block consists of a BIRD element, a hard 180° pulse, and a data acquisition window, with small delays (ca. 20 μs) flanking the hard 180° proton pulse set to refocus the chemical shift. The first and last chunks are half in size (*at*/2*n*) relative to the rest of the chunks (*at*/*n*). Phase cycling: *ϕ*_1_=[1 3]_4_, *ϕ*_2_=[0 2], *ϕ*_3_=[0 2]_8_, *ϕ*_4_=[0 2]_16_, *ϕ*_5_=[0 1]_2_, *ϕ*_6_=[1 2]_2_, *ϕ*_7_=[2 3]_2_, *ϕ*_R_={1 3 1 3 (3 1 3 1)_2_ 1 3 1 3 3 1 3 1 (1 3 1 3)_2_ 3 1 3 1}, all other pulses are of phase 0 (for the explicit phase table, see Table S1).

Figure 2 illustrates the application of the new real-time pure shift method to ^1^H-^13^C correlated spectra. The conventional gHSQC spectrum (Figure 2 a) of d(+)-fucose shows multiplet structure in the ^1^H frequency (*F*_2_) dimension; the structure is collapsed to singlets in the pure shift spectrum (Figure 2 b) obtained using the real-time pure shift gHSQC sequence of Figure 1. The 1D projections onto the ^1^H (*F*_2_) axis show, as expected, that the singlets in the pure shift spectrum are more intense than the corresponding multiplets in the conventional HSQC. Peak heights increase by an average factor of 1.7 for doublets and 2.9 for multiplets. Linewidths in the pure shift spectrum are very similar to those in the conventional spectrum; although signal losses from imperfect pulses, mismatch between *τ* and ^1^*J*_CH_, and transverse relaxation should, in principle, lead to wider lines in the pure shift spectrum, for this example the degradation is negligible. Similar results were obtained for quinine (Supporting Information, Figure S3); in this case the wider range of ^13^C chemical shifts means that some degradation in performance is seen at the edges of the spectrum. Any discontinuities in the decoupled signal, such as those caused by *T*_2_ relaxation during the BIRD sequence element, mismatch between the BIRD timing and ^1^*J*_CH_, or a breakdown of the condition *n*≫(*at*×*J*_HH_), will lead to small *F*_2_ sidebands at multiples of *n*/*at*. In the current work, the level of these sideband artifacts is typically around 1 % (Figure S5).

**Figure 2:**
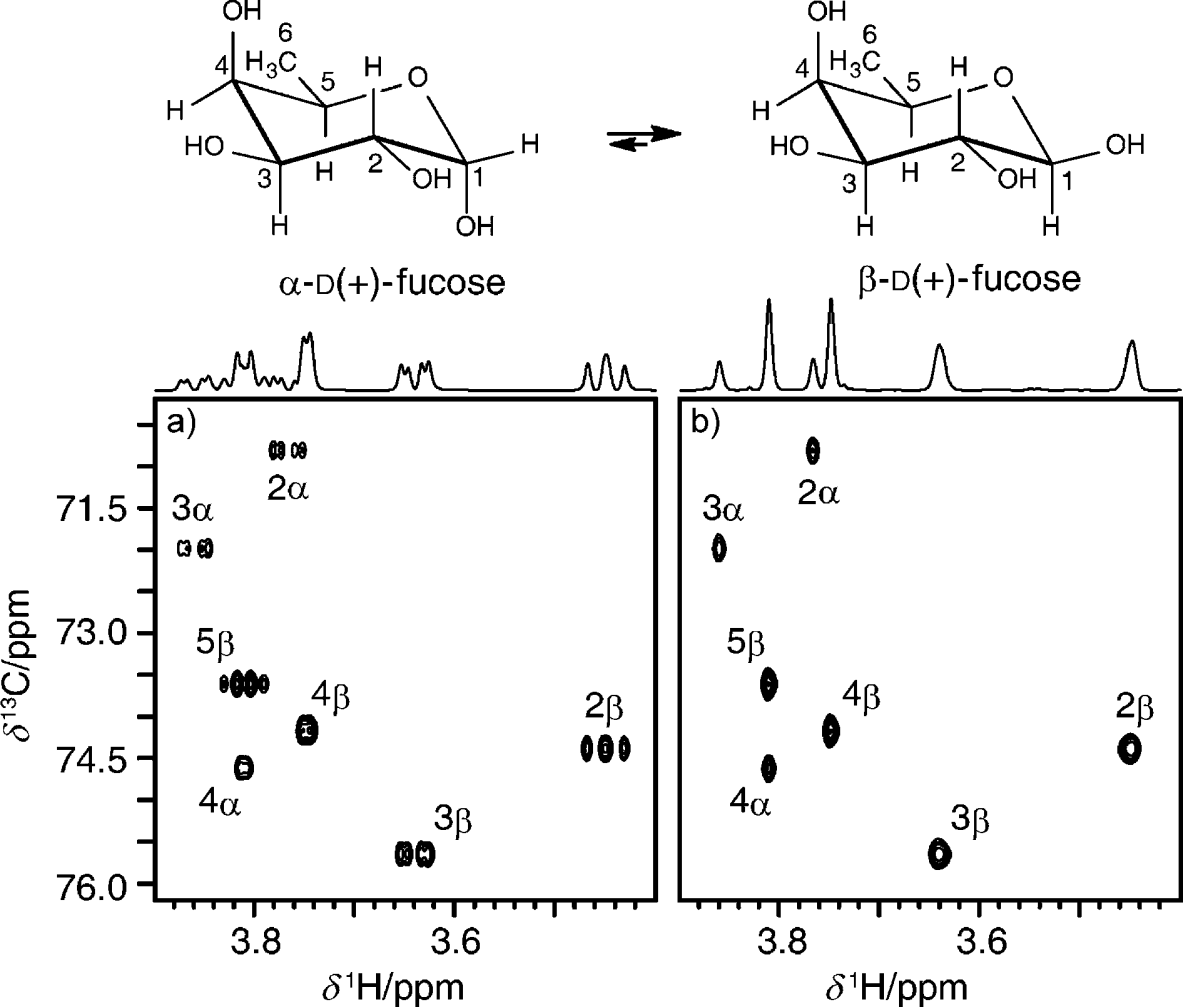
Selected regions (Indicated with dashed lines in the full spectra of Figure S1) of ^1^H-^13^C HSQC spectra of d(+)-fucose in D_2_O with TSP as internal reference: a) conventional gHSQC; b) real-time pure shift gHSQC. 1D traces are integral projections onto the *F*_2_ (^1^H) axis. Data were acquired, processed, and plotted with equivalent parameters, to allow quantitative comparison.

The proposed method is also applicable to ^1^H-^15^N correlation, either at natural abundance or in labeled systems where the labels are too far apart for ^15^N-^15^N coupling to be significant (as is generally the case in peptides and proteins). Figure 3 compares conventional and real-time pure shift HSQC spectra for ^15^N-labeled beta-amyloid peptide 1-42 (Aβ). The shaded region in the conventional HSQC spectrum (Figure 3 a) shows doublet resonances, which are collapsed to singlets in the pure shift HSQC spectrum (Figure 3 b). As shown in the spectra, this collapsing of multiplets again improves both the resolution and sensitivity of the signals. Overcrowding in the shaded region is reduced; for example, with overlap between the signals of isoleucines 32 and 41 much reduced in the pure shift spectrum.

**Figure 3:**
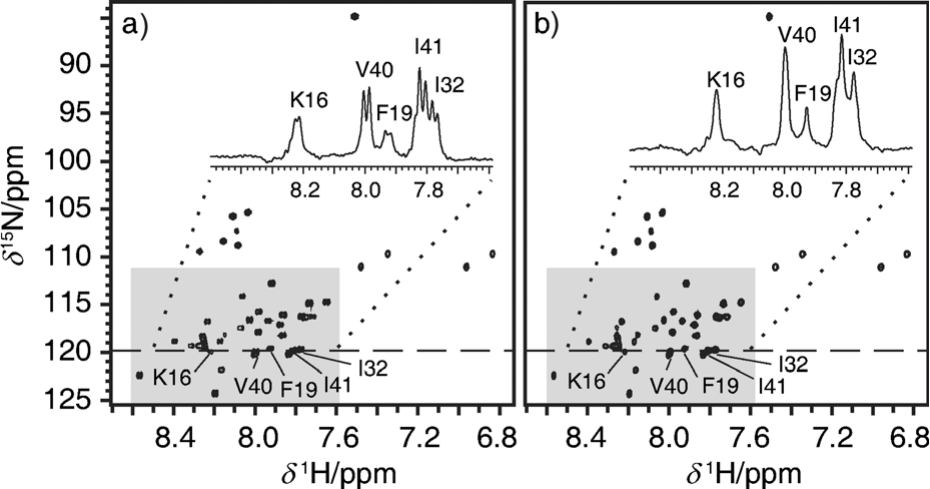
^1^H-^15^N HSQC spectra of ^15^N-labeled Aβ in [D_6_]dimethylsulfoxide containing H_2_O (5 %): a) conventional gHSQC; b) real-time pure shift gHSQC. 1D spectra are corresponding ^1^H traces at *δ*^15^N of 119.7 ppm. All data were acquired, processed, and plotted with equivalent parameters, to allow quantitative comparison. Expansions from shaded regions are shown in Figure S6.

In conclusion, the pure shift gHSQC method described here leads to complete collapse of multiplet resonances into singlets (except for nonequivalent methylene signals, which collapse to doublets). This homonuclear decoupling produces signals with increased intensity and better resolution, lowering detection limits, speeding up experiments, and improving the ability to distinguish between signals in complex spectra. This method is potentially well-suited to automated spectral analysis, as a single signal is seen for each distinct chemical site or correlation.

## Experimental Section

All experimental data were obtained using a Varian VNMRS 500 MHz spectrometer equipped with a triple resonance (^1^H/^13^C/^15^N) triple axis gradient probe of maximum *z* gradient 68.5 G cm^−1^, using GARP^5^ heteronuclear decoupling (γ*B*_2_/2π=4.2 kHz for ^13^C, 1.3 kHz for ^15^N) during data acquisition and BIP^28^ composite pulses. The spectra in Figure 2 were acquired at 20 °C using a 100 mM sample of d(+)-fucose in deuterium oxide, containing trimethylsilyl propanoic acid (TSP) as internal reference. The unusually high concentration was used in order to confirm that clean results are obtainable, with artifact signals at around the 1 % level. The following experimental and processing parameters were used: a hard 90° ^1^H pulse of duration 10.9 μs, a hard ^13^C 90^0^ pulse of duration 15.2 μs, a BIP composite 180° pulse (for Figure 2 b) of duration 125 μs and bandwidth 25 kHz; INEPT transfer delays *τ*=1.66 ms and BIRD delays 2*τ*=3.31 ms (equivalent to ^1^*J*_CH_=151 Hz); homospoil gradient pulses of 23.0 G cm^−1^ (G_1_) and 13.8 G cm^−1^ (G_3_) of durations 4.0 ms (δ_2_) and 2.4 ms (δ_4_), respectively; and coherence selection (CTP) gradients of 33.4 G cm^−1^ (G_2_) and 16.8 G cm^−1^ (G_4_) of durations 2.0 ms (δ_3_) and 1.0 ms (δ_5_), respectively; ^1^H spectral width (*sw*) was 3592.0 Hz; 4 transients were averaged for each of 2×512 free induction decays in which *t*_1_ was incremented to provide a ^13^C spectral width of 11 467.9 Hz (*sw1*) in the *F*_1_ dimension; total number of points (*np*) stored per FID was 4104, and for Figure 2 b *n* was 27. Data were zero filled to 16 384×8192, and Gaussian weighting was applied before double Fourier transformation. The total experiment times were 4.2 h for Figure 2 a and 4.4 h for Figure 2 b, the slightly greater duration for the latter arising from the 27 extra BIRD/180° elements in each FID.

For Figure 3, data were acquired at 25 °C using a solution of ^15^N-labeled Aβ in [D_6_]dimethylsulfoxide containing H_2_O (5 %). Experimental and processing parameters were: a hard 90° ^1^H pulse of duration 12.8 μs, a hard ^15^N 90° pulse of duration 44 μs, a BIP composite 180° pulse (for Figure 3 b) of duration 400 μs and bandwidth 6.5 kHz; INEPT transfer delays *τ*=2.78 ms and BIRD delays 2*τ*=5.56 ms (equivalent to ^1^*J*_NH_=90 Hz); homospoil gradient pulses of 23.0 G cm^−1^ (G_1_) and 13.8 G cm^−1^ (G_3_) of durations 4.0 ms (δ_2_) and 2.4 ms (δ_4_), respectively; and coherence selection (CTP) gradients of 33.4 G cm^−1^ (G_2_) and 16.9 G cm^−1^ (G_4_) of durations 2.0 ms (δ_3_) and 0.4 ms (δ_5_), respectively; ^1^H spectral width (*sw*) was 10.0 kHz; 32 transients were averaged for each of 2×64 free induction decays in which *t*_1_ was incremented to provide a ^15^N spectral width of 3.0 kHz (*sw1*) in the *F*_1_ dimension; number of points (*np*) sampled per FID was 4096, and for Figure 3 b *n* was 8. Data were zero filled to 16 384×512 and then Fourier transformed without weighting. The total experiment time was approximately 2.7 h in each case.

## Supplementary material

As a service to our authors and readers, this journal provides supporting information supplied by the authors. Such materials are peer reviewed and may be re-organized for online delivery, but are not copy-edited or typeset. Technical support issues arising from supporting information (other than missing files) should be addressed to the authors.
