# Supplementary material for: Simultaneously Enhancing Spectral Resolution and Sensitivity in Heteronuclear Correlation NMR Spectroscopy
Source: Angew Chem Int Ed Engl. 2013 Sep 6;52(44):11616–9. doi: 10.1002/anie.201305709 (PMC4065349; doi:10.1002/anie.201305709)
Supplement: Supplementary file 1 [file anie0052-11616-SD1.pdf]

Angewandte Chemie
Eine Zeitschrift der Gesellschaft Deutscher Chemiker

Supporting Information
© Wiley-VCH 2013
69451 Weinheim, Germany

Simultaneously Enhancing Spectral Resolution and Sensitivity in Heteronuclear Correlation NMR Spectroscopy**

*Liladhar Paudel, Ralph W. Adams, Péter Király, Juan A. Aguilar, Mohammadali Foroozandeh, Matthew J. Cliff, Mathias Nilsson, Péter Sándor, Jonathan P. Waltho, and Gareth A. Morris**

anie_201305709_sm_miscellaneous_information.pdf

Supplementary Information

**Table S1:** Explicit phase cycling for the real-time pure shift gHSQC pulse sequence of Figure 1 of the paper
ϕ_1={1,1,1,1,3,3,3,3}
ϕ_2={0,2}
ϕ_3={0,0,0,0,0,0,0,0,2,2,2,2,2,2,2,2}
ϕ_4={0,0,0,0,0,0,0,0,0,0,0,0,0,0,0,0,2,2,2,2,2,2,2,2,2,2,2,2,2,2,2,2}
ϕ_5={0,0,1,1}
ϕ_6={1,1,2,2}
ϕ_7={2,2,3,3}
ϕ_R={1,3,1,3,3,1,3,1,3,1,3,1,1,3,1,3,3,1,3,1,1,3,1,3,1,3,1,3,3,1,3,1}

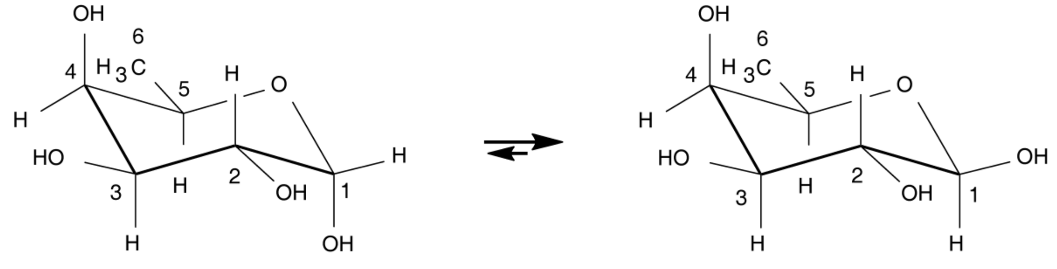

α-D(+)-Fucose β-D(+)-Fucose

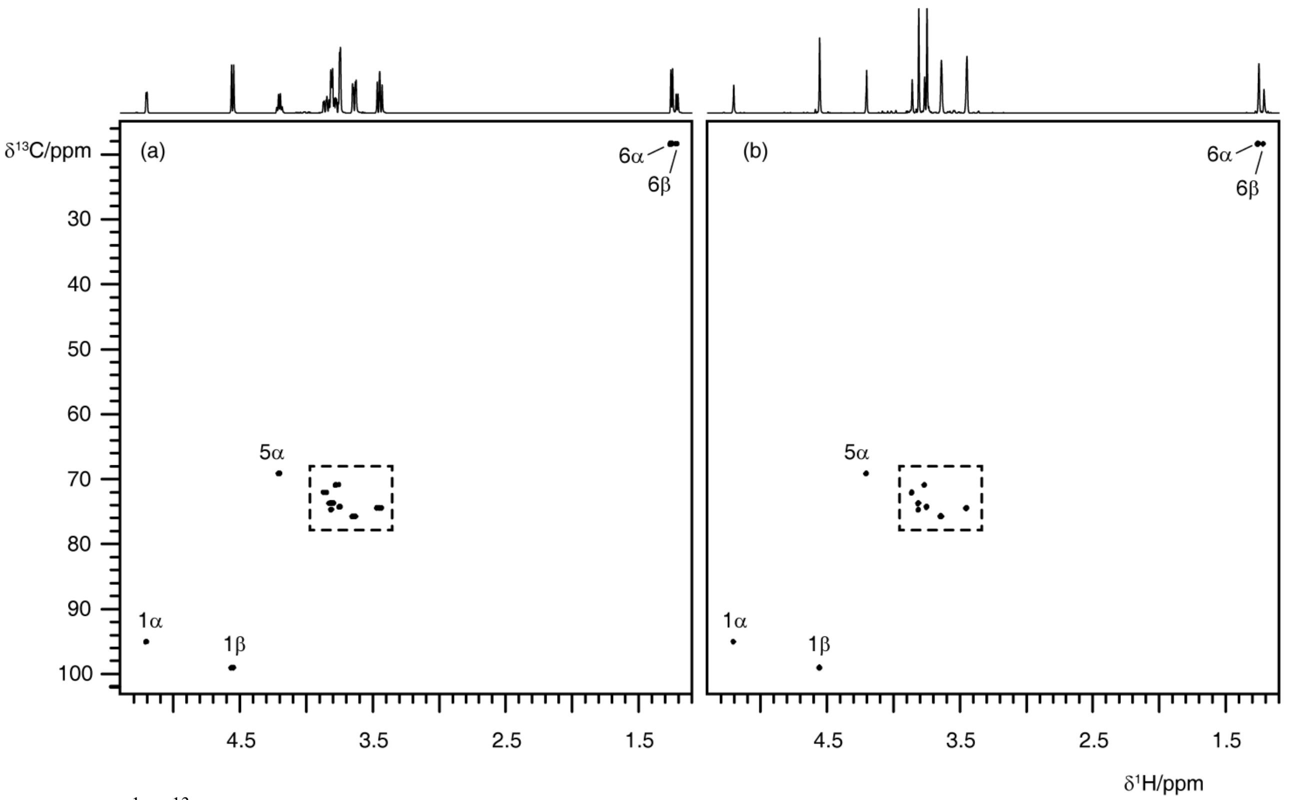

Figure S1. ^{1}H-^{13}C HSQC spectra (full view) of D(+)-fucose in D_2O with TSP as internal reference: (a) conventional HSQC and (b) real-time pure shift HSQC. Insets indicated with dashed lines are shown in Figure 2 of the paper. 1D traces are integral projections onto the F_2 (^{1}H) axis. Data were acquired, processed and plotted with equivalent parameters, to allow quantitative comparision (For detail, see Experimental Section of the paper).

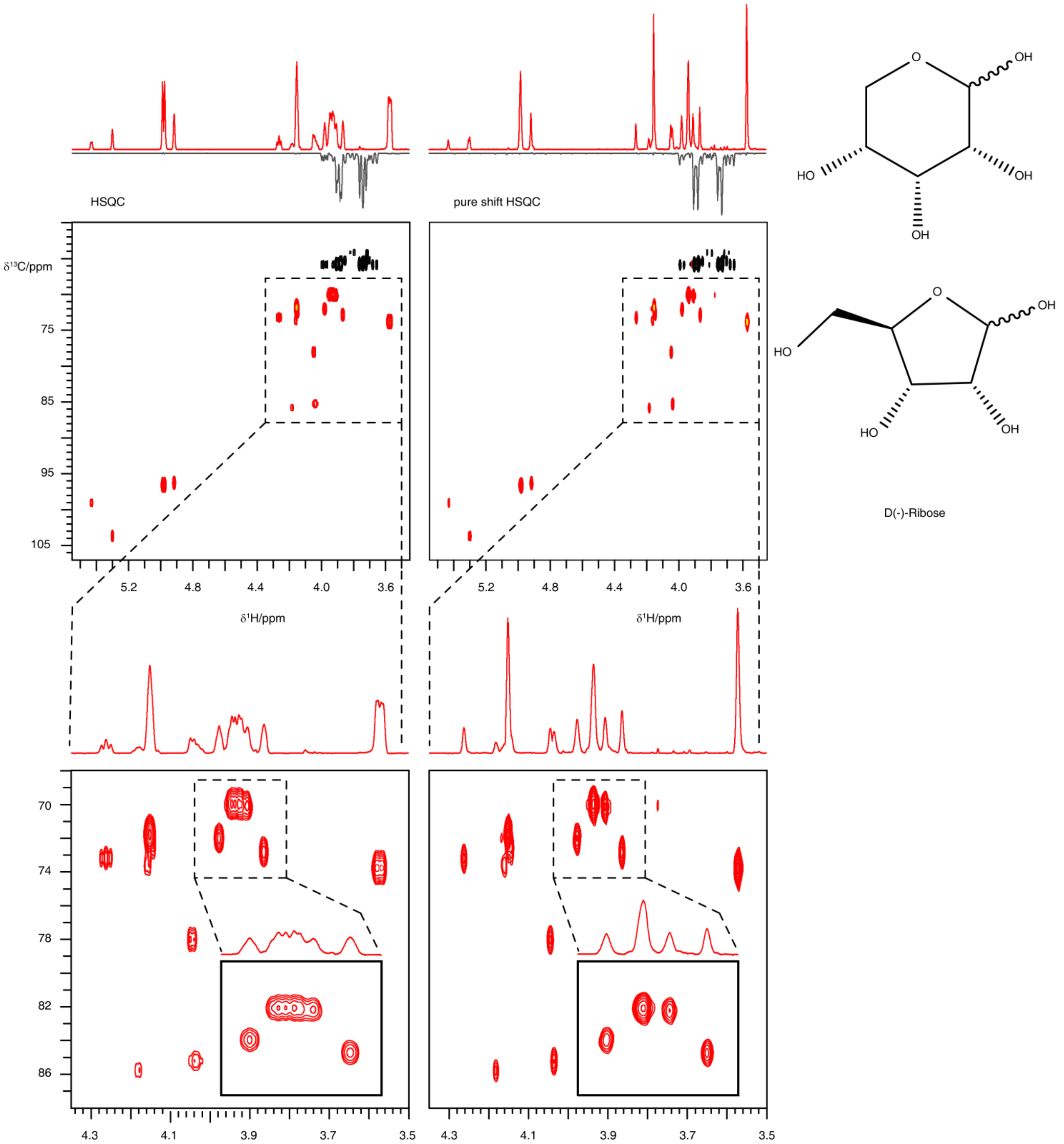

Figure S2. Conventional (left) and real-time pure shift (right) HSQC spectra of D(-)-ribose in D_2O. Spectra in top panel show full view and those in bottom panel show a selected region (indicated with dashed lines). Spectra were collected, processed and plotted with equivalent parameters. The sample concentration was 100 mM, and TSP was added as internal chemical shift reference. 16 transients were accumulated for each of 2×64 t_1 increments. Number of points sampled was 2816, and n was 16.

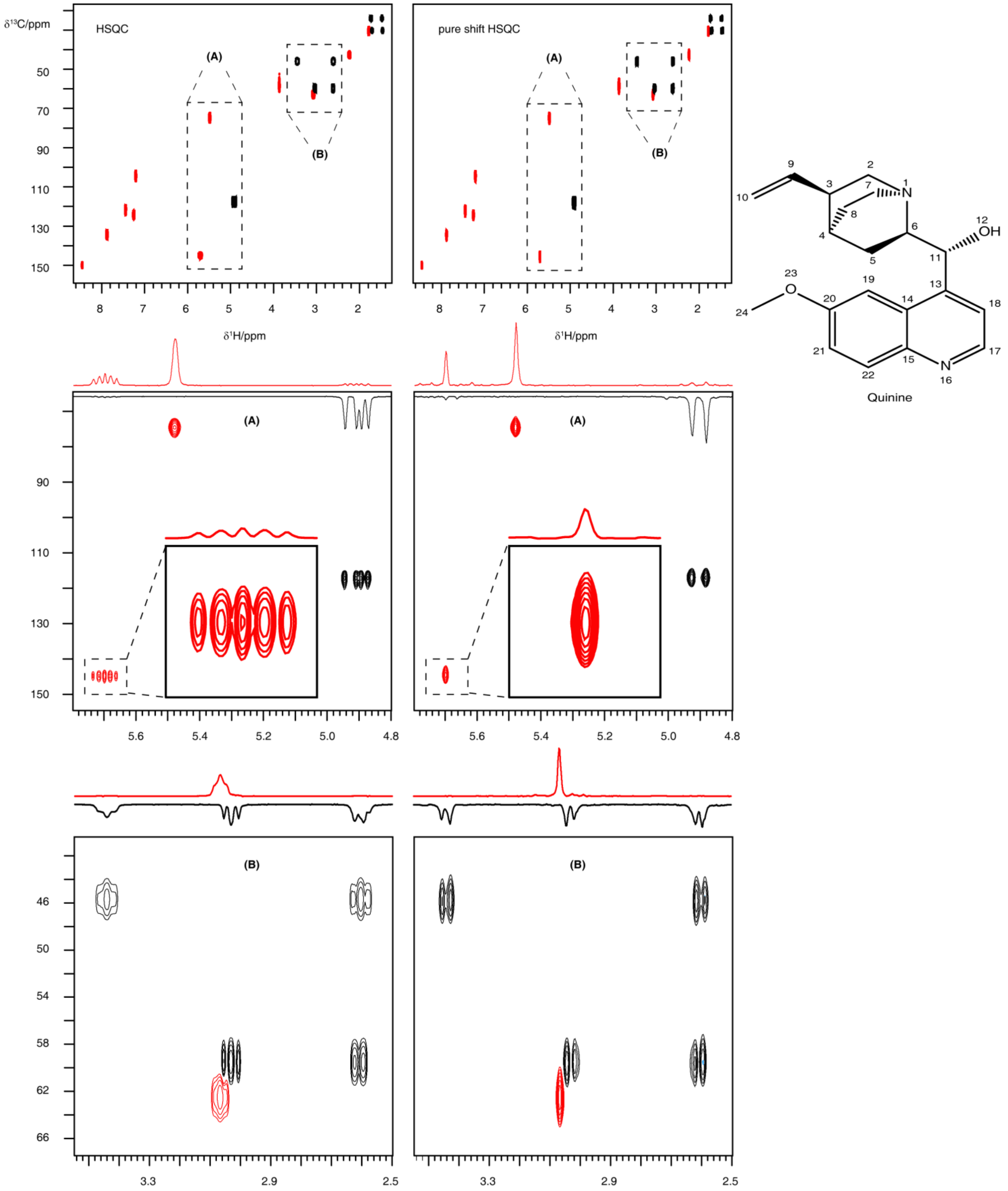

Figure S3. Conventional (left) and real-time pure shift (right) HSQC spectra of quinine in $CDCl_3$. Spectra in top panel show full view and those in middle and bottom panels show view from two selected regions (indicated with dashed lines). Spectra were collected, processed and plotted with equivalent parameters. The sample concentration was 90 mM, and TMS was added as internal chemical shift reference. 16 transients were accumulated for each of 2 × 64 t_1 increments. Number of points sampled was 4096, and *n* was 16.

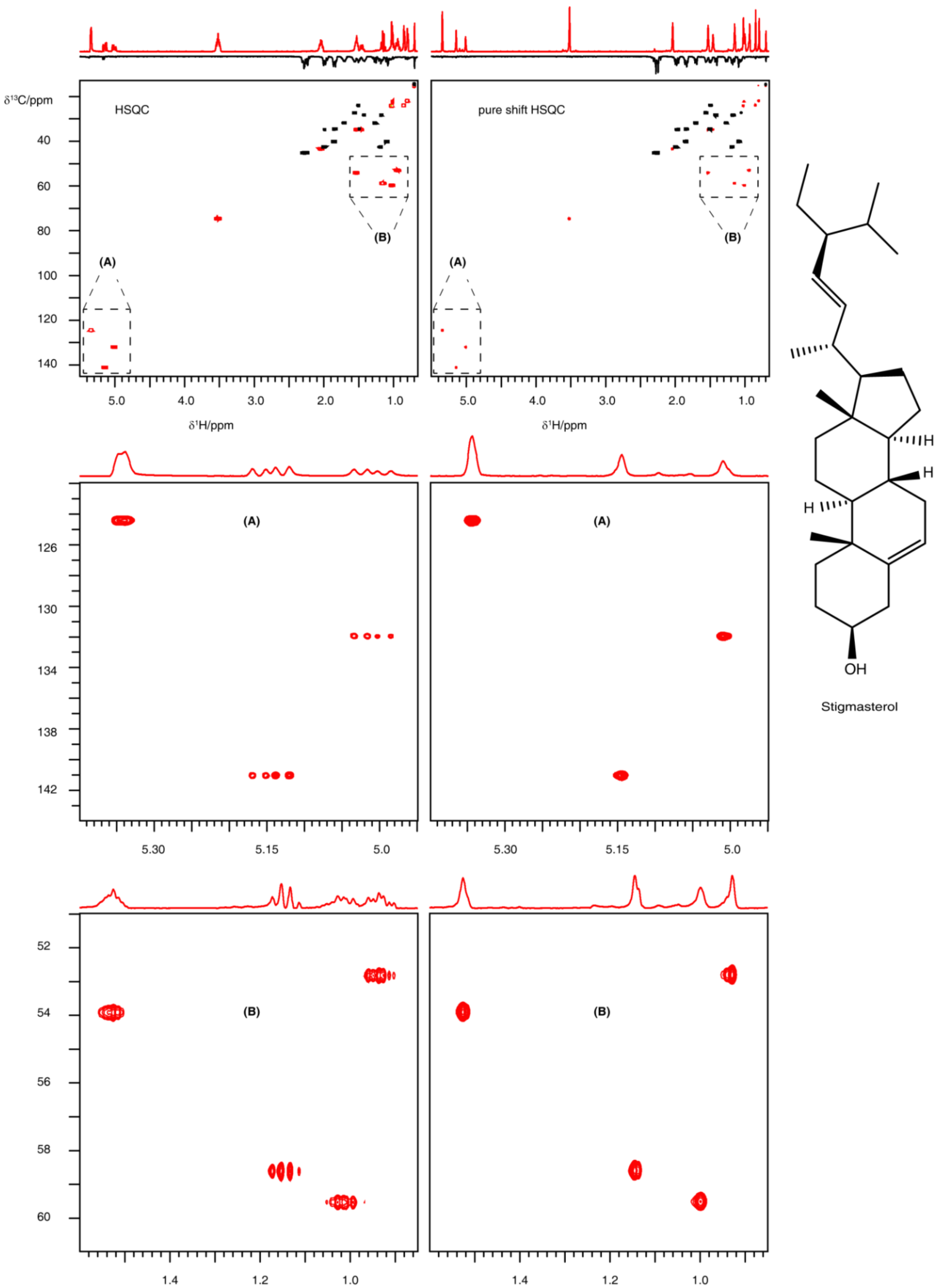

Figure S4. Conventional (left) and real-time pure shift (right) HSQC spectra of stigmasterol in $CDCl_3$. Spectra in top panel show full view and those in middle and bottom panels show view from two selected regions (indicated with dashed lines). Spectra were collected, processed and plotted with equivalent parameters. The sample concentration was 50 mM, and TMS was added as internal chemical shift reference. 4 transients were accumulated for each of 2 × 512 t_1 increments. Number of points sampled was 4104, and n was 27.

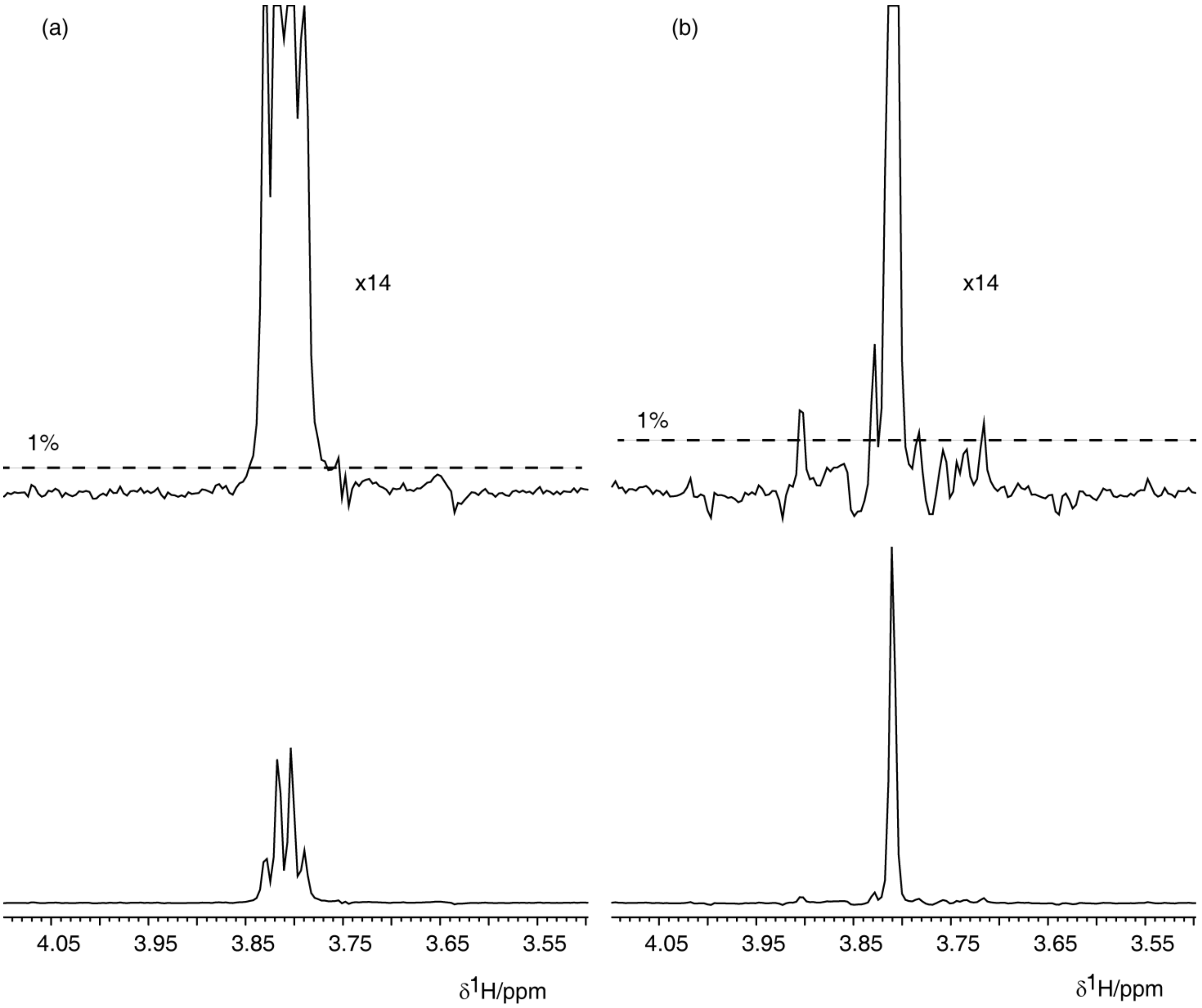

Figure S5. Vertically expanded ^{1}H traces at $\delta^{13}C$ of 73.6 ppm (chemical shift of 5β carbon) for the comparison of artifact levels in conventional and real-time pure shift HSQC spectra. Traces were taken from the (a) conventional HSQC and (b) real-time pure shift HSQC spectra of D(+)-fucose of Figure S1. Spectra from each panel were plotted at same vertical scale; the vertical scale of each spectrum in the top panel is 14 times that of corresponding spectrum in the bottom panel; 1% of the parent peak height of the main signal is indicated for the spectra in top panel.

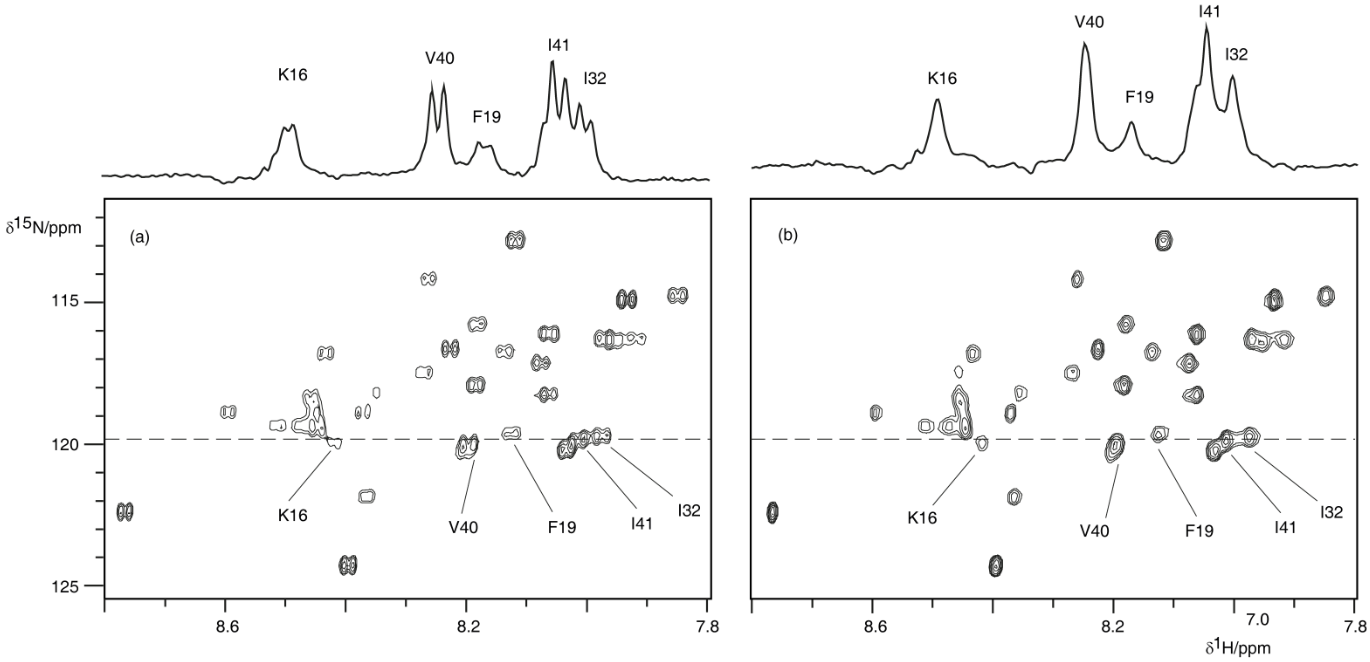

Figure S6. Shaded regions of Figure 3 of the paper: (a) conventional HSQC and (b) real-time HSQC. As described in Figure 3 of the paper, the 1D spectra shown at the top of 2D spectra are corresponding ^{1}H traces for δ^{15}N of 119.7 ppm. Spectra were collected, processed and plotted with equivalent parameters; for detail, see Experimental Section of the paper.

```
/*Pulse Sequence Code for real-time pure shift gHSQC using BIRD*/
/*--------------------------------------------------------------------------------------------------------------------------
Developed By NMR Group
School of Chemistry
University of Manchester
United Kingdom
May 2013

----------------------------------------------------------------------
User's Guide for experimental setup:
----------------------------------------------------------------------
1. BIRD = 'n' selects conventional gHSQC
----------------------------------------------------------------------
2.  BIRD = 'y' selects real-time pure shift gHSQC (gHSQC-BIRD)
        Three options within BIRD are:
                BIRDmode ='h' selects hard 13C inversion pulse during BIRD // large off-resonance effect, not recommended
                BIRDmode ='b' selects BIP 13C inversion pulse during BIRD
                BIRDmode ='w' selects a pair of wurst adiabatic 13C inversion pulses during BIRD
                For all np should be integer submultiple of npoints
                Users control chunking time using npoints so that np/npoints is an integer
                Note:
                chunk_time=npoints/(2*sw)=at/cycles
                cycles=np/npoints, an integer
                at=np/(2*sw)=cycles*npoints/(2*sw)=cycles*chunk_time
-----------------------------------------------------------------------------------------------------------------------------*/

#include <standard.h>
//#include <chempack.h>

/*-------------------------------------------
Phase tables for Varian gHSQC
-------------------------------------------*/

static int     ph1[4]  = {1,1,3,3},                          //v1   - proton 90 at the end of first inept
               ph2[2]  = {0,2},                      //v2   - X 90 at the end of first inept
               ph3[8]  = {0,0,0,0,2,2,2,2},                  //v3   - proton 90 in 2nd inept
               ph4[16] = {0,0,0,0,0,0,0,0,2,2,2,2,2,2,2,2},          //v4   - X 90 in 2nd inept
               ph5[16] = {1,3,3,1,3,1,1,3,3,1,1,3,1,3,3,1};          //oph
/*-------------------------------------------
Phase tables for rtgHSQC-BIRD
-------------------------------------------*/
static int     ph11[8]  = {1,1,1,1,3,3,3,3},                           //v1
               ph12[2]  = {0,2},                           //v2
               ph13[16] = {0,0,0,0,0,0,0,0,2,2,2,2,2,2,2,2},            //v3
               ph14[32] = {0,0,0,0,0,0,0,0,0,0,0,0,0,0,0,0,2,2,2,2,2,2,2,2,2,2,2,2,2,2,2,2},   //v4
               ph15[32] = {1,3,1,3,3,1,3,1,3,1,3,1,1,3,1,3,3,1,3,1,1,3,1,3,1,3,1,3,3,1,3,1};   //oph
static int          ph17[4]  = {0,0,1,1},               //v7 - 1st 90 of bird and the hard 180 refocusing
               ph18[4]  = {1,1,2,2},                //v8 - simpulse 180 of bird
               ph19[4]  = {2,2,3,3};                //v9 - 2nd 90 of bird
pulsesequence()
{
/*-------------------------------------------------------
 DECLARE AND LOAD VARIABLES
-------------------------------------------------------*/
//HSQC part

double  evolcorr=2.0*pw+4.0e-6,
        tau = 1.0/(4.0*(getval("j1xh"))),
        taug=2.0*tau,
        mult = getval("mult");

int     phase1 = (int)(getval("phase")+0.5),
        ZZgsign=1.0,
        icosel;
//BIRD
double
        rof3=getval("rof3"),            //delay for receiver off - can be zero if ddrpm='r'
        tauA=getval("tauA"),            //compensation for tauB and tauC
        tauB=getval("tauB"),            //effect of rof2
```

```
        tauC=getval("tauC"),            //effect of alfa
        tBal=getval("tBal"),            //supports inova console if ~1/(fb*1.3); set to zero in VNMRS system
        pwr_XBIP = getval("pwr_XBIP"),
        pwr_HBIP = getval("pwr_HBIP"),
        pw_XBIP = getval("pw_XBIP"),
        pw_HBIP = getval("pw_HBIP"),
        npoints=getval("npoints"),              // npoints should be an integer multiple of np
        cycles=np/npoints;
        cycles = (double)((int)((cycles)));
        initval(cycles,v20);

char            shp_HBIP[MAXSTR],
        shp_XBIP[MAXSTR];
        getstr("shp_HBIP",shp_HBIP);
        getstr("shp_XBIP",shp_XBIP);

//extensions for AD
double          pwx180 = getval("pwx180"),
        pwxlvl180 = getval("pwxlvl180"),
        pwx180r = getval("pwx180r"),
        pwxlvl180r = getval("pwxlvl180r");

char    pwx180ad[MAXSTR],
        pwx180adR[MAXSTR];
        getstr("pwx180ad", pwx180ad);
        getstr("pwx180adR", pwx180adR);
//gradients
double          gtE = getval("gtE"),            //HSQC encoding
        gzlvlE = getval("gzlvlE"),
        gstab = getval("gstab"),
        gtD = getval("gtD"),            //HSQC decoding
        gzlvlD = getval("gzlvlD"),
        hsglvl = getval("hsglvl"),
        hsgt = getval("hsgt"),
        hsgstab = getval("hsgstab");

char            BIRD[MAXSTR],                   // Flag to choose gHSQC/rtgHSQC-BIRD ('n'/'y')
        BIRDmode[MAXSTR];       //Flag to choose hard/bip/wurst2i ('h'/'b'/'w')13C inversion pulse within BIRD
        getstr("BIRD",BIRD);
        getstr("BIRDmode",BIRDmode);

char            sspul[MAXSTR],
        PFGflg[MAXSTR];
        getstr("sspul",sspul);
        getstr("PFGflg",PFGflg);

//evolcorr and mult declarations
        evolcorr = 2*pw+4.0e-6;
        if (mult > 0.5)
        taug = 2*tau;
        else
        taug = gtE + gstab + 2*GRADIENT_DELAY;
        ZZgsign=-1;
        if (mult == 2) ZZgsign=1;
        icosel = 1;

//setup the phase cycle
        assign(ct,v10);


if (BIRD[0]=='n')
{
//gHSQC phases
        settable(t1,4,ph1);
        settable(t2,2,ph2);
        settable(t3,8,ph3);
        settable(t4,16,ph4);
        settable(t5,16,ph5);
}
```

```
else
{
//rtgHSQC-BIRD phases
        settable(t1,8,ph11);
        settable(t2,2,ph12);
        settable(t3,16,ph13);
        settable(t4,32,ph14);
        settable(t5,32,ph15);
        settable(t7,4,ph17);
        settable(t8,4,ph18);
        settable(t9,4,ph19);
        getelem(t7, v10, v7);
        getelem(t8, v10, v8);
        getelem(t9, v10, v9);
}

        getelem(t1, v10, v1);
        getelem(t2, v10, v2);
        getelem(t3, v10, v3);
        getelem(t4, v10, v4);
        getelem(t5, v10, oph);


        initval(2.0*(double)(((int)(d2*getval("sw1")+0.5)%2)),v5);
        if ((phase1 == 2) || (phase1 == 5))
        icosel = -1;

        add(v2,v5,v2);
        add(oph,v5,oph);

/* BEGIN PULSE SEQUENCE */
status(A);

        if (sspul[A] == 'y')
        {
        if (PFGflg[A] == 'y')
        {
        obspower(tpwr);
        delay(5.0e-5);
       zgradpulse(hsglvl,hsgt);
       rgpulse(pw,zero,rof1,rof1);
       zgradpulse(hsglvl,hsgt);
        }
        else
        {
       obspower(tpwr-12);
        delay(5.0e-5);
       rgpulse(500*pw,zero,rof1,rof1);
       rgpulse(500*pw,one,rof1,rof1);
        }
        }

        obspower(tpwr);
        decpower(pwxlvl);
        txphase(zero);
       decphase(zero);
        obsoffset(tof);
        decoffset(dof);

        delay(d1);
        delay(5.0e-5);

status(B);

/****** null flag starts here *****/

     if (getflag("nullflg"))
     {
       rgpulse(0.5*pw,zero,rof1,rof1);
```

```
         txphase(zero);
         delay(2.0*tau);
         simpulse(2.0*pw,2.0*pwx,zero,zero,rof1,rof1);
         txphase(two);
        delay(2.0*tau);
        rgpulse(1.5*pw,two,rof1,rof1);
         txphase(zero);
         zgradpulse(hsglvl,hsgt);
        delay(hsgstab);
      }

/****************************gHSQC or gHSQC part of pure shift starts here *************************/

         rgpulse(pw,zero,0.0,0.0);
         delay(tau);
         simpulse(2.0*pw,2.0*pwx,zero,zero,rof1,rof1);
         txphase(v1);
         delay(tau);
         rgpulse(pw,v1,rof1,rof1);

         zgradpulse(hsglvl,2.0*hsgt);
         decphase(v2);
         delay(hsgstab);

         decrgpulse(pwx, v2, rof1, 2.0e-6);
         txphase(zero);
         decphase(zero);

         delay(d2/2.0);                         // First half of t1 evolution
         rgpulse(2.0*pw,zero,2.0e-6,2.0e-6);
         delay(d2/2.0);                         // Second half of t1 evolution

         zgradpulse(gzlvlE,gtE);
         delay(taug - gtE - 2.0*GRADIENT_DELAY);
         simpulse(mult*pw,2.0*pwx,zero,zero,rof1,rof1);
         delay(taug + evolcorr);

         decrgpulse(pwx,v4,2.0e-6,rof1);
         zgradpulse(ZZgsign*0.6*hsglvl,1.2*hsgt);
         txphase(v3);
         delay(hsgstab);

         rgpulse(pw,v3,rof1,rof1);
         delay(tau - (2.0*pw/PI) - 2.0*rof1);
         simpulse(2.0*pw,2.0*pwx,zero,zero,rof1,rof1);

         zgradpulse(icosel*gzlvlD,gtD);
         decpower(dpwr);
         delay(tau - gtD - 2.0*GRADIENT_DELAY - POWER_DELAY);

/*********************************gHSQC part stops and BIRD Acquisition starts here**************************/

         delay(tBal);
         //filter delay (Hoult) for inova; adjust tBal manually for the same effect
         //delay(1.0/(getval("fb")*1.3))
         //tBal sets to zero in VNMRS system

if (BIRD[0]=='y')
{
setacqmode(WACQ|NZ);      //use this line only for vnmrs console; comment this out in inova


         obsblank();
         delay(rof2);
         startacq(alfa);
}
/*------------------------------------------------------------------------------------------------------------------------
               Observe the 1st half chunk
------------------------------------------------------------------------------------------------------------------------*/
```

```
if (BIRD[0]=='y')
{
status(C);
        acquire(npoints/2.0,1.0/sw);
        rcvroff();
status(B);
        obspower(tpwr);
        txphase(v7);

/*-----------------------------------------------------------------------
Using hard 13C inversion pulse in BIRD
-------------------------------------------------------------------------*/
if (BIRDmode[0]== 'h')
{
        rgpulse(pw,v7,rof1,rof1);
        decpower(pwxlvl);
        delay(2.0*tau);
        simpulse(2.0*pw,2.0*pwx,v8,v8,rof1,rof1);
        decpower(dpwr);
        delay(2.0*tau);
        rgpulse(pw,v9,rof1,rof1);
}

/*--------------------------------------------------------------------------
Using BIP 13C inversion pulse in BIRD
----------------------------------------------------------------------------*/
if (BIRDmode[0]== 'b')
{
        rgpulse(pw,v7,rof1,rof1);
        if (pwr_HBIP!=tpwr)  obspower(pwr_HBIP);
        if (pwr_XBIP!=pwxlvl) decpower(pwr_XBIP); else decpower(pwxlvl);
        delay(2.0*tau);
        simshaped_pulse(shp_HBIP,shp_XBIP,pw_HBIP,pw_XBIP,v8,v8,rof1,rof1);
        if (pwr_HBIP!=tpwr) obspower(tpwr);
        decpower(dpwr);
        delay(2.0*tau);
        rgpulse(pw,v9,rof1,rof1);
}

/*--------------------------------------------------------------------------
Using a pair of wurst2i adiabatic 13C inversion pulses in BIRD
----------------------------------------------------------------------------*/
if (BIRDmode[0]== 'w')
{
        rgpulse(pw,v7,rof1,rof1);
        if (pwr_HBIP!=tpwr)  obspower(pwr_HBIP);
        if (pwxlvl180!=pwxlvl) decpower(pwxlvl180); else decpower(pwxlvl);
        txphase(v8); decphase(v8);
        delay(2.0*tau);
        decshaped_pulse(pwx180ad, pwx180, v8, rof1, rof1);
        shaped_pulse(shp_HBIP,pw_HBIP,v8,rof1,rof1);
        if (pwr_HBIP!=tpwr) obspower(tpwr);
        txphase(v9);
        delay(2.0*tau);
        decshaped_pulse(pwx180adR, pwx180, v8, rof1, rof1);
        decpower(dpwr);
        rgpulse(pw,v9,rof1,rof1);
}

        txphase(v7);
        delay(tauA);
        rgpulse(pw*2.0,v7,rof1,rof1);    // hard 180 degree refocusing pulse
        obsblank();
        delay(tauB);
        rcvron();    //this includes rof3
        delay(tauC);

decr(v20);
```

```
/*-----------------------------------------------------------------------------------------------------------------------------
                Loops for more chunks
-----------------------------------------------------------------------------------------------------------------------------*/

starthardloop(v20);
status(C);
        acquire(npoints,1.0/sw);
        rcvroff();

status(B);
        obspower(tpwr);
        txphase(v7);

/*------------------------------------------------------------------------
Using hard 13C inversion pulse in BIRD
------------------------------------------------------------------------*/
if (BIRDmode[0]== 'h')
{
        rgpulse(pw,v7,rof1,rof1);
        decpower(pwxlvl);
        delay(2.0*tau);
        simpulse(2.0*pw,2.0*pwx,v8,v8,rof1,rof1);
        decpower(dpwr);
        delay(2.0*tau);
        rgpulse(pw,v9,rof1,rof1);
}

/*--------------------------------------------------------------------------
Using BIP 13C inversion pulse in BIRD
--------------------------------------------------------------------------*/
if (BIRDmode[0]== 'b')
{
        rgpulse(pw,v7,rof1,rof1);
        if (pwr_HBIP!=tpwr)  obspower(pwr_HBIP);
        if (pwr_XBIP!=pwxlvl) decpower(pwr_XBIP); else decpower(pwxlvl);
        delay(2.0*tau);
        simshaped_pulse(shp_HBIP,shp_XBIP,pw_HBIP,pw_XBIP,v8,v8,rof1,rof1);
        if (pwr_HBIP!=tpwr) obspower(tpwr);
        decpower(dpwr);
        delay(2.0*tau);
        rgpulse(pw,v9,rof1,rof1);
}

/*-------------------------------------------------------------------------
Using a pair of wurst2i adiabatic 13C inversion pulses in BIRD
--------------------------------------------------------------------------*/
if (BIRDmode[0]== 'w')
{
        rgpulse(pw,v7,rof1,rof1);
        if (pwr_HBIP!=tpwr)  obspower(pwr_HBIP);
        if (pwxlvl180!=pwxlvl) decpower(pwxlvl180); else decpower(pwxlvl);
        txphase(v8); decphase(v8);
        delay(2.0*tau);
        decshaped_pulse(pwx180ad, pwx180, v8, rof1, rof1);
        shaped_pulse(shp_HBIP,pw_HBIP,v8,rof1,rof1);
        if (pwr_HBIP!=tpwr) obspower(tpwr);
        txphase(v9);
        delay(2.0*tau);
        decshaped_pulse(pwx180adR, pwx180, v8, rof1, rof1);
        decpower(dpwr);
        rgpulse(pw,v9,rof1,rof1);
}

        txphase(v7);
        delay(tauA);
        rgpulse(pw*2.0,v7,rof1,rof1);                // hard 180 degree refocusing pulse
        obsblank();
        delay(tauB);
        rcvron();                           //this includes rof3
```

```
        delay(tauC);

endhardloop();

/*-----------------------------------------------------------------------------------------------------------------------------
                Acquisition of last half chunk
-----------------------------------------------------------------------------------------------------------------------------*/
status(C);
        acquire(npoints/2.0,1.0/sw);
        rcvroff();
        endacq();
        incr(v20);
}

                                        /****** BIRD ends here for all *****/


/************************ ACQ for conventional gHSQC ***********************************/

else
{
status(C);
}

}
/********************* PULSE SEQUENCE ENDS HERE****************************************/
```
